# A dimeric sesquiterpene, gochnatiolide A

**DOI:** 10.1107/S1600536809042743

**Published:** 2009-10-23

**Authors:** Hui-Ping Xiong, Zhi-Jun Wu, Dong-Sheng Chen, Wan-Sheng Chen

**Affiliations:** a Department of Mathematics and Physics, Shanghai University of Electric Power, Shanghai 200090, People’s Republic of China; b Department of Pharmacy, Changzheng Hospital, Second Military Medical University, Shanghai 200003, People’s Republic of China

## Abstract

The title compound [systematic name: 5′a-hydroxy-1′,3,6,8′-tetrakis(methylene)-3a,4,5,5′,5′a,6,6′,6a,7,7′,7′a,8′,9a,9b,10′a,10′b-hexadecahydrospiro[azuleno[4,5-*b*]furan-9(2*H*),3′-[3*H*]benz[1,8]azuleno[4,5-*b*]furan]-2,2′,8,9′(1′*H*,3*H*,4′*H*)-tetrone acetone 0.92-solvate], C_30_H_30_O_7_·0.92C_3_H_6_O, is a dimeric sequiterpene formed by a cyclohexane system connecting two monomeric sesquiterpene lactone units of dehydro­zaluzanin C. It was isolated from *Ainsliaea henryi*.

## Related literature

For similar compounds and background information, see: *Chinese Materia Medica* (2007 ▶); Bohlmann & Zdero (1979 ▶); Bohlmann *et al.* (1981 ▶, 1982 ▶, 1983 ▶, 1984 ▶, 1986 ▶). For the pharmacological activity of a related compound, see: Wu *et al.* (2008 ▶). 
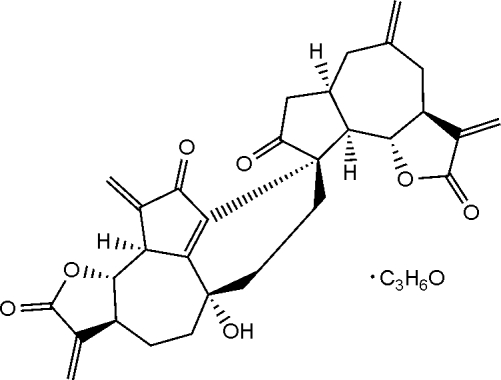


## Experimental

### 

#### Crystal data

C_30_H_30_O_7_·0.92C_3_H_6_O
*M* *_r_* = 555.94
Orthorhombic, 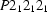

*a* = 8.709 (4) Å
*b* = 12.652 (6) Å
*c* = 25.890 (12) Å
*V* = 2853 (2) Å^3^
*Z* = 4
Mo *K*α radiation
μ = 0.09 mm^−1^
*T* = 294 K
0.15 × 0.10 × 0.08 mm

#### Data collection

Bruker SMART APEX CCD area-detector diffractometer
Absorption correction: multi-scan (*SADABS*; Sheldrick, 1996 ▶) *T*_min_ = 0.986, *T*_max_ = 0.993
11789 measured reflections
2864 independent reflections
1873 reflections with *I* > 2σ(*I*)
*R*_int_ = 0.078

#### Refinement

*R*[*F* ^2^ > 2σ(*F* ^2^)] = 0.047
*wR*(*F* ^2^) = 0.119
*S* = 0.93
2864 reflections
401 parameters
128 restraints
H-atom parameters constrained
Δρ_max_ = 0.32 e Å^−3^
Δρ_min_ = −0.12 e Å^−3^

### 

Data collection: *SMART* (Bruker, 2005 ▶); cell refinement: *SAINT* (Bruker, 2005 ▶); data reduction: *SAINT*; program(s) used to solve structure: *SHELXS97* (Sheldrick, 2008 ▶); program(s) used to refine structure: *SHELXL97* (Sheldrick, 2008 ▶); molecular graphics: *SHELXTL* (Sheldrick, 2008 ▶); software used to prepare material for publication: *SHELXL97*.

## Supplementary Material

Crystal structure: contains datablocks I, global. DOI: 10.1107/S1600536809042743/pk2191sup1.cif

Structure factors: contains datablocks I. DOI: 10.1107/S1600536809042743/pk2191Isup2.hkl

Additional supplementary materials:  crystallographic information; 3D view; checkCIF report

## Figures and Tables

**Fig. 1.**
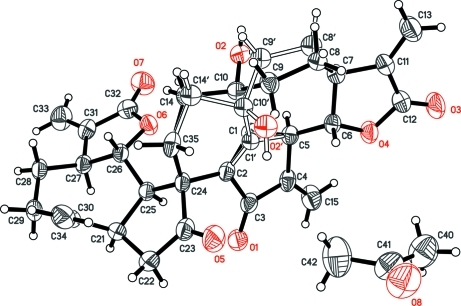
The molecular structure of (I), showing the atom-labelling scheme with displacement ellipsoids drawn at the 30% probability level. H atoms are shown as small spheres of arbitrary radius.

**Fig. 2.**
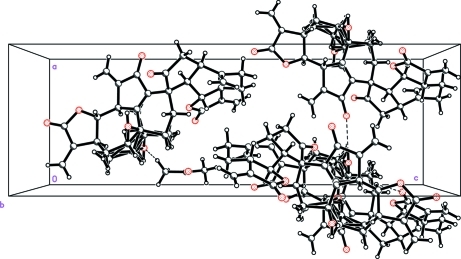
The molecular packing of (I), viewed along the a axis.

## supplementary crystallographic information

### Comment

*Ainsliaea henryi Diels* is mainly distributed in south-west of China. The whole plant of *Ainsliaea henryi* has been used in Chinese folk medicine to treat cough, asthma and lumbago (Editorial committee of Chinese Mateia Medica, 2007). The chemical constituents of this plant have not been reported previously. Our chemical investigation of this plant for bioactive components resulted in the isolation of the title compound (I), which was previously obtained from the South American species, *Gochnatia paniculata* (Bohlmann *et al.*, 1983) and *Gochnatia polymorpha* (Bohlmann *et al.*, 1986). This kind of dimeric sesquiterpene has exhibited a remarkable inhibitory activity against the production of nitric oxide in RAW264.7 (Mouse leukaemic monocyte macrophage cell line) stimulated by LPS (Lipopolysaccharide) (Wu *et al.*, 2008).

The molecular struture of (I) is shown in Fig.1; bond lengths and angles are within normal ranges. Gochnatiolide A is a dimeric sequiterpene which was derived from two molecules of the compound dehydrozaluzanin C. The dehydrozaluzanin C molecule is composed of a seven-membered ring and two five-membered rings (A ring atoms C1—C5; B ring atoms C1—C10; C ring atoms C6/C7/C11/C12/O4). The connections between the two dehydrozaluzanin C molecules are the bonds C2—C24, and C10—C14—C35—C24. Ring A adopts an envelope conformation, ring B adopts a badly distorted chair conformation, while ring C exhibits an envelope conformation.

### Experimental

Dry powders (5 kg) of the whole plant of *Ainsliaea henryi* were refluxed for 1 h with 95% ethanol (50*L*) three times. After removal of the ethanol under reduced pressure, the extract was suspended in water and then partitioned with petroleum ether, chloroform, ethyl acetate and n-butanol. The chloroform soluble fraction (30 g) was subjected to silica gel column chromatography using gradient elution (petroleum ether/acetone, 15:1 to 2:1, *v*/*v*). Gochnatiolide A was obtained from the fraction eluted by petroleum ether/acetone (5:1). Single crystals suitable for X-ray diffraction analysis were obtained by slow evaporation from petroleum ether/acetone (1:1) after two weeks at room temperature.

### Refinement

The hydroxyl H atoms attached at O2 was located in a difference Fourier map and isotropically refined using a riding model with O—H distance 0.82 Å. The remaining H atoms were placed in calculated positions with C—H distances in the range 0.93–0.98 Å. The *U*ĩso values were set equal to 1.5*U*eq (C) for methyl H atoms and 1.5*U*eq(C) for the remaining H atoms. Friedel pairs were merged before the final refinement as there is no significant anomalous dispersion.  As a consequence the absolute configuration of the compound is unknown.The stereochemistry of the title compound is known from the literature (Bohlmann *et al.* (1983)).Its structure was elucidated by highfield 1H-NMR spectroscopy. We have also confirmed its structure by 1H, 13C, 2DNMR spectroscopy.

A number of restraints (128 in total) were required the ensure that the geometry (SADI and SAME in SHELXL-97) and displacement parameters (SIMU in SHELXL-97) retained chemically and physically reasonable values during the refinement.

### Crystal data

**Table:**

C_30_H_30_O_7_·0.92C_3_H_6_O	*F*(000) = 1182
*M**_r_* = 555.94	*D*_x_ = 1.294 Mg m^−^^3^
Orthorhombic, *P*2_1_2_1_2_1_	Mo *K*α radiation, λ = 0.71073 Å
Hall symbol: P 2ac 2ab	Cell parameters from 1001 reflections
*a* = 8.709 (4) Å	θ = 2.5–24.3°
*b* = 12.652 (6) Å	µ = 0.09 mm^−^^1^
*c* = 25.890 (12) Å	*T* = 294 K
*V* = 2853 (2) Å^3^	Block, colourless
*Z* = 4	0.15 × 0.10 × 0.08 mm

### Data collection

**Table:**

Bruker SMART APEX CCD area-detector diffractometer	2864 independent reflections
Radiation source: sealed tube	1873 reflections with *I* > 2σ(*I*)
graphite	*R*_int_ = 0.078
φ and ω scans	θ_max_ = 25.0°, θ_min_ = 1.6°
Absorption correction: multi-scan (*SADABS*; Sheldrick, 1996)	*h* = −7→10
*T*_min_ = 0.986, *T*_max_ = 0.993	*k* = −15→14
11789 measured reflections	*l* = −30→30

### Refinement

**Table:**

Refinement on *F*^2^	Secondary atom site location: difference Fourier map
Least-squares matrix: full	Hydrogen site location: inferred from neighbouring sites
*R*[*F*^2^ > 2σ(*F*^2^)] = 0.047	H-atom parameters constrained
*wR*(*F*^2^) = 0.119	*w* = 1/[σ^2^(*F*_o_^2^) + (0.0673*P*)^2^] where *P* = (*F*_o_^2^ + 2*F*_c_^2^)/3
*S* = 0.93	(Δ/σ)_max_ < 0.001
2864 reflections	Δρ_max_ = 0.32 e Å^−^^3^
401 parameters	Δρ_min_ = −0.12 e Å^−^^3^
128 restraints	Extinction correction: *SHELXL*, Fc^*^=kFc[1+0.001xFc^2^λ^3^/sin(2θ)]^-1/4^
0 constraints	Extinction coefficient: 0.0034 (11)
Primary atom site location: structure-invariant direct methods	

### Special details

**Table:**

Geometry. All e.s.d.'s (except the e.s.d. in the dihedral angle between two l.s. planes) are estimated using the full covariance matrix. The cell e.s.d.'s are taken into account individually in the estimation of e.s.d.'s in distances, angles and torsion angles; correlations between e.s.d.'s in cell parameters are only used when they are defined by crystal symmetry. An approximate (isotropic) treatment of cell e.s.d.'s is used for estimating e.s.d.'s involving l.s. planes.
Refinement. Refinement of *F*^2^ against ALL reflections. The weighted *R*-factor *wR* and goodness of fit *S* are based on *F*^2^, conventional *R*-factors *R* are based on *F*, with *F* set to zero for negative *F*^2^. The threshold expression of *F*^2^ >2σ(*F*^2^) is used only for calculating *R*-factors(gt) *etc*. and is not relevant to the choice of reflections for refinement. *R*-factors based on *F*^2^ are statistically about twice as large as those based on *F*, and *R*- factors based on ALL data will be even larger.

### Fractional atomic coordinates and isotropic or equivalent isotropic displacement parameters (Å^2^)

**Table:**

	*x*	*y*	*z*	*U*_iso_*/*U*_eq_	Occ. (<1)
O1	0.4589 (3)	0.8430 (3)	0.72146 (11)	0.0859 (10)	
O3	−0.0138 (4)	0.9219 (3)	0.95361 (11)	0.1010 (12)	
O4	0.0768 (3)	0.8957 (2)	0.87468 (10)	0.0704 (8)	
O5	0.3123 (4)	0.6000 (3)	0.68984 (12)	0.0936 (10)	
O6	0.0973 (3)	0.9794 (2)	0.61271 (10)	0.0648 (8)	
O7	0.0044 (4)	1.1419 (3)	0.60213 (13)	0.0963 (11)	
C2	0.1914 (4)	0.8169 (3)	0.70590 (13)	0.0511 (9)	
C3	0.3257 (5)	0.8549 (3)	0.73401 (15)	0.0609 (10)	
C4	0.2677 (5)	0.9100 (3)	0.78106 (14)	0.0609 (11)	
C5	0.0944 (4)	0.9005 (3)	0.78111 (13)	0.0523 (9)	
H5	0.0503	0.9716	0.7791	0.063*	
C6	0.0263 (4)	0.8446 (3)	0.82731 (13)	0.0510 (9)	
H6	0.0602	0.7707	0.8274	0.061*	
C7	−0.1479 (5)	0.8484 (4)	0.82852 (14)	0.0612 (10)	
H7	−0.1816	0.9131	0.8111	0.073*	
C11	−0.1797 (5)	0.8596 (4)	0.88515 (15)	0.0695 (12)	
C12	−0.0371 (5)	0.8938 (4)	0.91013 (17)	0.0737 (12)	
C13	−0.3092 (7)	0.8448 (5)	0.91028 (19)	0.0967 (16)	
H13A	−0.3133	0.8562	0.9457	0.116*	
H13B	−0.3965	0.8230	0.8926	0.116*	
C8	−0.2239 (6)	0.7527 (4)	0.80139 (18)	0.0570 (14)	0.808 (5)
H8A	−0.2150	0.6919	0.8240	0.068*	0.808 (5)
H8B	−0.3325	0.7677	0.7974	0.068*	0.808 (5)
C9	−0.1589 (6)	0.7225 (4)	0.74833 (17)	0.0584 (13)	0.808 (5)
H9A	−0.2389	0.6861	0.7292	0.070*	0.808 (5)
H9B	−0.0761	0.6724	0.7537	0.070*	0.808 (5)
C10	−0.0979 (5)	0.8128 (4)	0.71442 (17)	0.0512 (11)	0.808 (5)
O2	−0.1932 (4)	0.9031 (3)	0.71949 (13)	0.0660 (10)	0.808 (5)
H2	−0.2835	0.8847	0.7197	0.099*	0.808 (5)
C14	−0.0889 (5)	0.7804 (5)	0.65696 (19)	0.0609 (17)	0.808 (5)
H14A	−0.1830	0.7440	0.6478	0.073*	0.808 (5)
H14B	−0.0833	0.8440	0.6361	0.073*	0.808 (5)
C1	0.0602 (6)	0.8438 (4)	0.7319 (3)	0.0468 (15)	0.808 (5)
C8'	−0.263 (2)	0.7876 (19)	0.7957 (6)	0.062 (5)*	0.192 (5)
H8'1	−0.2629	0.7133	0.8049	0.075*	0.192 (5)
H8'2	−0.3661	0.8153	0.8008	0.075*	0.192 (5)
C9'	−0.214 (2)	0.8023 (17)	0.7405 (7)	0.060 (3)*	0.192 (5)
H9'1	−0.2103	0.8778	0.7340	0.073*	0.192 (5)
H9'2	−0.2944	0.7736	0.7188	0.073*	0.192 (5)
C10'	−0.0634 (19)	0.7563 (13)	0.7216 (7)	0.051 (3)*	0.192 (5)
O2'	−0.0418 (17)	0.6540 (11)	0.7438 (6)	0.070 (4)*	0.192 (5)
H2'	0.0503	0.6411	0.7456	0.106*	0.192 (5)
C14'	−0.102 (2)	0.754 (3)	0.6631 (7)	0.062 (4)*	0.192 (5)
H14C	−0.1885	0.7082	0.6555	0.074*	0.192 (5)
H14D	−0.1220	0.8243	0.6495	0.074*	0.192 (5)
C1'	0.081 (3)	0.817 (2)	0.7323 (14)	0.045 (4)*	0.192 (5)
C15	0.3579 (6)	0.9603 (4)	0.81350 (18)	0.0902 (17)	
H15A	0.4632	0.9626	0.8076	0.108*	
H15B	0.3165	0.9935	0.8423	0.108*	
C21	0.3861 (4)	0.7640 (3)	0.58050 (14)	0.0600 (10)	
H21	0.4744	0.8079	0.5709	0.072*	
C22	0.4426 (5)	0.6809 (4)	0.61919 (16)	0.0775 (13)	
H22A	0.4597	0.6139	0.6019	0.093*	
H22B	0.5383	0.7034	0.6349	0.093*	
C23	0.3214 (5)	0.6699 (4)	0.65901 (15)	0.0693 (11)	
C24	0.2052 (4)	0.7607 (3)	0.65507 (13)	0.0519 (9)	
C25	0.2759 (4)	0.8337 (3)	0.61335 (13)	0.0511 (9)	
H25	0.3408	0.8844	0.6318	0.061*	
C26	0.1675 (4)	0.8979 (3)	0.58093 (12)	0.0509 (9)	
H26	0.0862	0.8510	0.5682	0.061*	
C27	0.2387 (5)	0.9549 (3)	0.53460 (13)	0.0558 (10)	
H27	0.3469	0.9696	0.5422	0.067*	
C28	0.2292 (5)	0.8915 (3)	0.48538 (14)	0.0612 (10)	
H28A	0.2586	0.9365	0.4567	0.073*	
H28B	0.1232	0.8706	0.4800	0.073*	
C29	0.3292 (5)	0.7929 (4)	0.48453 (14)	0.0665 (11)	
H29A	0.3035	0.7524	0.4539	0.080*	
H29B	0.4355	0.8149	0.4812	0.080*	
C30	0.3170 (4)	0.7206 (4)	0.53086 (14)	0.0585 (10)	
C31	0.1522 (5)	1.0569 (3)	0.53462 (16)	0.0658 (11)	
C32	0.0762 (5)	1.0678 (4)	0.58487 (18)	0.0683 (12)	
C33	0.1407 (7)	1.1302 (4)	0.49896 (19)	0.0936 (16)	
H33A	0.0833	1.1908	0.5053	0.112*	
H33B	0.1899	1.1215	0.4674	0.112*	
C34	0.2586 (5)	0.6260 (4)	0.5270 (2)	0.0837 (14)	
H34A	0.2233	0.6017	0.4953	0.100*	
H34B	0.2522	0.5829	0.5561	0.100*	
C35	0.0480 (5)	0.7089 (3)	0.64304 (13)	0.0637 (11)	
H35A	0.0434	0.6920	0.6065	0.076*	
H35B	0.0397	0.6433	0.6622	0.076*	
O8	0.3631 (7)	0.4623 (4)	0.8854 (2)	0.146 (2)	0.919 (7)
C40	0.3646 (10)	0.6279 (6)	0.9215 (3)	0.133 (3)	0.919 (7)
H40A	0.3417	0.5887	0.9523	0.199*	0.919 (7)
H40B	0.2852	0.6791	0.9155	0.199*	0.919 (7)
H40C	0.4612	0.6634	0.9255	0.199*	0.919 (7)
C41	0.3730 (8)	0.5566 (5)	0.8781 (3)	0.104 (2)	0.919 (7)
C42	0.3737 (11)	0.6047 (7)	0.8249 (3)	0.153 (3)	0.919 (7)
H42A	0.4679	0.5870	0.8077	0.230*	0.919 (7)
H42B	0.3649	0.6801	0.8276	0.230*	0.919 (7)
H42C	0.2886	0.5775	0.8054	0.230*	0.919 (7)

### Atomic displacement parameters (Å^2^)

**Table:**

	*U*^11^	*U*^22^	*U*^33^	*U*^12^	*U*^13^	*U*^23^
O1	0.0530 (19)	0.142 (3)	0.0630 (18)	−0.0116 (19)	−0.0036 (14)	−0.0044 (19)
O3	0.105 (3)	0.149 (3)	0.0488 (18)	−0.008 (2)	0.0050 (17)	−0.024 (2)
O4	0.0734 (18)	0.090 (2)	0.0480 (15)	−0.0099 (17)	−0.0003 (14)	−0.0065 (15)
O5	0.120 (3)	0.088 (2)	0.072 (2)	0.024 (2)	0.009 (2)	0.0258 (19)
O6	0.0758 (18)	0.0645 (17)	0.0542 (16)	0.0076 (15)	0.0078 (14)	−0.0041 (15)
O7	0.114 (3)	0.071 (2)	0.104 (2)	0.021 (2)	0.007 (2)	−0.0157 (19)
C2	0.055 (2)	0.057 (2)	0.041 (2)	−0.0060 (19)	−0.0039 (18)	0.0063 (17)
C3	0.052 (2)	0.083 (3)	0.048 (2)	−0.016 (2)	−0.006 (2)	0.008 (2)
C4	0.069 (3)	0.070 (3)	0.044 (2)	−0.024 (2)	−0.0062 (19)	0.000 (2)
C5	0.058 (2)	0.051 (2)	0.048 (2)	−0.0062 (18)	−0.0026 (18)	−0.0016 (18)
C6	0.061 (2)	0.049 (2)	0.043 (2)	−0.0018 (18)	−0.0023 (18)	−0.0045 (17)
C7	0.059 (2)	0.076 (3)	0.049 (2)	0.006 (2)	0.0055 (19)	0.002 (2)
C11	0.068 (3)	0.088 (3)	0.053 (2)	0.003 (3)	0.012 (2)	−0.003 (2)
C12	0.078 (3)	0.088 (3)	0.055 (3)	0.006 (3)	0.010 (2)	−0.006 (2)
C13	0.091 (4)	0.128 (4)	0.071 (3)	−0.009 (3)	0.017 (3)	−0.014 (3)
C8	0.044 (3)	0.074 (3)	0.053 (3)	−0.002 (2)	0.002 (2)	0.002 (2)
C9	0.059 (3)	0.065 (3)	0.051 (2)	−0.013 (2)	0.004 (2)	−0.004 (2)
C10	0.047 (2)	0.056 (2)	0.051 (2)	−0.003 (2)	−0.0003 (19)	0.000 (2)
O2	0.060 (2)	0.076 (2)	0.062 (2)	0.0098 (19)	−0.0026 (18)	−0.0013 (19)
C14	0.051 (3)	0.084 (4)	0.048 (3)	−0.024 (3)	0.003 (2)	−0.002 (3)
C1	0.049 (3)	0.047 (3)	0.044 (2)	−0.003 (3)	−0.004 (2)	0.005 (3)
C15	0.085 (3)	0.119 (4)	0.067 (3)	−0.038 (3)	0.000 (3)	−0.014 (3)
C21	0.049 (2)	0.078 (3)	0.053 (2)	0.008 (2)	0.0035 (18)	0.002 (2)
C22	0.065 (3)	0.106 (4)	0.061 (3)	0.016 (3)	0.000 (2)	0.014 (2)
C23	0.082 (3)	0.077 (3)	0.048 (2)	0.012 (3)	−0.005 (2)	0.008 (2)
C24	0.059 (2)	0.062 (2)	0.0348 (18)	−0.0041 (19)	−0.0005 (17)	0.0052 (17)
C25	0.052 (2)	0.060 (2)	0.041 (2)	−0.0039 (18)	−0.0013 (17)	0.0007 (18)
C26	0.052 (2)	0.058 (2)	0.0423 (19)	−0.006 (2)	−0.0009 (17)	−0.0030 (18)
C27	0.059 (2)	0.064 (2)	0.044 (2)	−0.0039 (19)	−0.0001 (18)	0.0052 (19)
C28	0.066 (3)	0.078 (3)	0.039 (2)	−0.008 (2)	−0.0010 (18)	0.000 (2)
C29	0.068 (3)	0.089 (3)	0.042 (2)	−0.002 (3)	0.002 (2)	−0.007 (2)
C30	0.052 (2)	0.070 (3)	0.053 (2)	0.009 (2)	0.0046 (19)	−0.006 (2)
C31	0.074 (3)	0.067 (3)	0.056 (3)	−0.007 (2)	−0.010 (2)	0.008 (2)
C32	0.067 (3)	0.062 (3)	0.076 (3)	0.001 (2)	−0.007 (2)	−0.007 (2)
C33	0.122 (4)	0.074 (3)	0.084 (3)	0.009 (3)	−0.005 (3)	0.017 (3)
C34	0.085 (3)	0.083 (3)	0.083 (3)	0.010 (3)	0.007 (3)	−0.014 (3)
C35	0.081 (3)	0.067 (3)	0.043 (2)	−0.018 (2)	−0.010 (2)	0.0013 (19)
O8	0.191 (6)	0.105 (4)	0.143 (4)	0.033 (4)	−0.016 (4)	−0.010 (3)
C40	0.156 (7)	0.126 (6)	0.117 (5)	−0.007 (5)	−0.026 (5)	−0.016 (5)
C41	0.131 (6)	0.074 (4)	0.106 (5)	0.024 (4)	−0.027 (4)	−0.022 (4)
C42	0.208 (9)	0.138 (7)	0.114 (5)	−0.043 (7)	0.002 (6)	−0.016 (5)

### Geometric parameters (Å, °)

**Table:**

O1—C3	1.214 (5)	C10'—C1'	1.499 (10)
O3—C12	1.198 (5)	C10'—C14'	1.554 (17)
O4—C12	1.351 (5)	O2'—H2'	0.8200
O4—C6	1.454 (4)	C14'—C35	1.518 (11)
O5—C23	1.193 (5)	C14'—H14C	0.9700
O6—C32	1.344 (5)	C14'—H14D	0.9700
O6—C26	1.454 (4)	C15—H15A	0.9300
O7—C32	1.212 (5)	C15—H15B	0.9300
C2—C1'	1.18 (3)	C21—C30	1.522 (5)
C2—C1	1.370 (8)	C21—C22	1.533 (6)
C2—C3	1.459 (5)	C21—C25	1.556 (5)
C2—C24	1.500 (5)	C21—H21	0.9800
C3—C4	1.492 (6)	C22—C23	1.482 (6)
C4—C15	1.315 (5)	C22—H22A	0.9700
C4—C5	1.514 (5)	C22—H22B	0.9700
C5—C1	1.491 (8)	C23—C24	1.535 (6)
C5—C6	1.511 (5)	C24—C25	1.548 (5)
C5—C1'	1.65 (3)	C24—C35	1.549 (5)
C5—H5	0.9800	C25—C26	1.502 (5)
C6—C7	1.518 (5)	C25—H25	0.9800
C6—H6	0.9800	C26—C27	1.531 (5)
C7—C11	1.499 (5)	C26—H26	0.9800
C7—C8'	1.525 (11)	C27—C31	1.494 (6)
C7—C8	1.548 (6)	C27—C28	1.509 (5)
C7—H7	0.9800	C27—H27	0.9800
C11—C13	1.315 (6)	C28—C29	1.522 (6)
C11—C12	1.465 (7)	C28—H28A	0.9700
C13—H13A	0.9300	C28—H28B	0.9700
C13—H13B	0.9300	C29—C30	1.512 (5)
C8—C9	1.534 (6)	C29—H29A	0.9700
C8—H8A	0.9700	C29—H29B	0.9700
C8—H8B	0.9700	C30—C34	1.304 (6)
C9—C10	1.536 (6)	C31—C33	1.312 (6)
C9—H9A	0.9700	C31—C32	1.466 (6)
C9—H9B	0.9700	C33—H33A	0.9300
C10—O2	1.418 (6)	C33—H33B	0.9300
C10—C1	1.502 (6)	C34—H34A	0.9300
C10—C14	1.545 (6)	C34—H34B	0.9300
O2—H2	0.8200	C35—H35A	0.9700
C14—C35	1.539 (6)	C35—H35B	0.9700
C14—H14A	0.9700	O8—C41	1.211 (7)
C14—H14B	0.9700	C40—C41	1.441 (8)
C8'—C9'	1.505 (17)	C40—H40A	0.9600
C8'—H8'1	0.9700	C40—H40B	0.9600
C8'—H8'2	0.9700	C40—H40C	0.9600
C9'—C10'	1.513 (16)	C41—C42	1.507 (9)
C9'—H9'1	0.9700	C42—H42A	0.9600
C9'—H9'2	0.9700	C42—H42B	0.9600
C10'—O2'	1.427 (15)	C42—H42C	0.9600
			
C12—O4—C6	110.1 (3)	H14C—C14'—H14D	109.7
C32—O6—C26	110.2 (3)	C2—C1'—C10'	125 (2)
C1'—C2—C3	111.3 (13)	C2—C1'—C5	112.8 (12)
C1—C2—C3	109.9 (4)	C10'—C1'—C5	122 (2)
C1'—C2—C24	125.1 (13)	C4—C15—H15A	120.0
C1—C2—C24	128.1 (4)	C4—C15—H15B	120.0
C3—C2—C24	121.9 (3)	H15A—C15—H15B	120.0
O1—C3—C2	126.3 (4)	C30—C21—C22	115.5 (4)
O1—C3—C4	126.9 (4)	C30—C21—C25	114.9 (3)
C2—C3—C4	106.8 (3)	C22—C21—C25	103.3 (3)
C15—C4—C3	123.0 (4)	C30—C21—H21	107.6
C15—C4—C5	129.3 (4)	C22—C21—H21	107.6
C3—C4—C5	107.6 (3)	C25—C21—H21	107.6
C1—C5—C6	111.9 (3)	C23—C22—C21	106.9 (3)
C1—C5—C4	103.7 (4)	C23—C22—H22A	110.3
C6—C5—C4	115.4 (3)	C21—C22—H22A	110.3
C6—C5—C1'	106.2 (11)	C23—C22—H22B	110.3
C4—C5—C1'	97.0 (8)	C21—C22—H22B	110.3
C1—C5—H5	108.5	H22A—C22—H22B	108.6
C6—C5—H5	108.5	O5—C23—C22	125.6 (4)
C4—C5—H5	108.5	O5—C23—C24	123.7 (4)
C1'—C5—H5	121.2	C22—C23—C24	110.7 (4)
O4—C6—C5	109.9 (3)	C2—C24—C23	110.4 (3)
O4—C6—C7	105.7 (3)	C2—C24—C25	111.2 (3)
C5—C6—C7	113.2 (3)	C23—C24—C25	103.3 (3)
O4—C6—H6	109.3	C2—C24—C35	107.8 (3)
C5—C6—H6	109.3	C23—C24—C35	106.2 (3)
C7—C6—H6	109.3	C25—C24—C35	117.6 (3)
C11—C7—C6	102.0 (3)	C26—C25—C24	117.6 (3)
C11—C7—C8'	118.1 (8)	C26—C25—C21	112.9 (3)
C6—C7—C8'	129.2 (10)	C24—C25—C21	106.8 (3)
C11—C7—C8	116.0 (4)	C26—C25—H25	106.3
C6—C7—C8	113.1 (4)	C24—C25—H25	106.3
C11—C7—H7	108.4	C21—C25—H25	106.3
C6—C7—H7	108.4	O6—C26—C25	109.4 (3)
C8'—C7—H7	88.1	O6—C26—C27	106.2 (3)
C8—C7—H7	108.4	C25—C26—C27	116.0 (3)
C13—C11—C12	123.4 (4)	O6—C26—H26	108.4
C13—C11—C7	129.0 (4)	C25—C26—H26	108.4
C12—C11—C7	107.6 (4)	C27—C26—H26	108.4
O3—C12—O4	120.6 (4)	C31—C27—C28	115.6 (3)
O3—C12—C11	130.2 (4)	C31—C27—C26	101.7 (3)
O4—C12—C11	109.1 (4)	C28—C27—C26	112.9 (3)
C11—C13—H13A	120.0	C31—C27—H27	108.8
C11—C13—H13B	120.0	C28—C27—H27	108.8
H13A—C13—H13B	120.0	C26—C27—H27	108.8
C9—C8—C7	116.3 (4)	C27—C28—C29	114.6 (3)
C9—C8—H8A	108.2	C27—C28—H28A	108.6
C7—C8—H8A	108.2	C29—C28—H28A	108.6
C9—C8—H8B	108.2	C27—C28—H28B	108.6
C7—C8—H8B	108.2	C29—C28—H28B	108.6
H8A—C8—H8B	107.4	H28A—C28—H28B	107.6
C8—C9—C10	117.0 (4)	C30—C29—C28	116.4 (3)
C8—C9—H9A	108.1	C30—C29—H29A	108.2
C10—C9—H9A	108.1	C28—C29—H29A	108.2
C8—C9—H9B	108.1	C30—C29—H29B	108.2
C10—C9—H9B	108.1	C28—C29—H29B	108.2
H9A—C9—H9B	107.3	H29A—C29—H29B	107.3
O2—C10—C1	107.3 (4)	C34—C30—C29	121.5 (4)
O2—C10—C9	110.1 (4)	C34—C30—C21	123.4 (4)
C1—C10—C9	109.8 (4)	C29—C30—C21	115.0 (4)
O2—C10—C14	109.4 (4)	C33—C31—C32	121.5 (4)
C1—C10—C14	108.3 (4)	C33—C31—C27	130.4 (4)
C9—C10—C14	111.8 (4)	C32—C31—C27	108.0 (3)
C35—C14—C10	114.8 (4)	O7—C32—O6	121.1 (4)
C35—C14—H14A	108.6	O7—C32—C31	129.3 (4)
C10—C14—H14A	108.6	O6—C32—C31	109.6 (4)
C35—C14—H14B	108.6	C31—C33—H33A	120.0
C10—C14—H14B	108.6	C31—C33—H33B	120.0
H14A—C14—H14B	107.5	H33A—C33—H33B	120.0
C2—C1—C5	111.9 (4)	C30—C34—H34A	120.0
C2—C1—C10	123.4 (5)	C30—C34—H34B	120.0
C5—C1—C10	124.5 (5)	H34A—C34—H34B	120.0
C9'—C8'—C7	106.1 (13)	C14'—C35—C24	122.2 (12)
C9'—C8'—H8'1	110.5	C14—C35—C24	112.9 (4)
C7—C8'—H8'1	110.5	C14'—C35—H35A	112.3
C9'—C8'—H8'2	110.5	C14—C35—H35A	109.0
C7—C8'—H8'2	110.5	C24—C35—H35A	109.0
H8'1—C8'—H8'2	108.7	C14'—C35—H35B	94.8
C8'—C9'—C10'	120.5 (16)	C14—C35—H35B	109.0
C8'—C9'—H9'1	107.2	C24—C35—H35B	109.0
C10'—C9'—H9'1	107.2	H35A—C35—H35B	107.8
C8'—C9'—H9'2	107.2	C41—C40—H40A	109.5
C10'—C9'—H9'2	107.2	C41—C40—H40B	109.5
H9'1—C9'—H9'2	106.8	H40A—C40—H40B	109.5
O2'—C10'—C1'	106.4 (15)	C41—C40—H40C	109.5
O2'—C10'—C9'	109.5 (14)	H40A—C40—H40C	109.5
C1'—C10'—C9'	117.9 (17)	H40B—C40—H40C	109.5
O2'—C10'—C14'	113.8 (16)	O8—C41—C40	119.4 (7)
C1'—C10'—C14'	111.8 (18)	O8—C41—C42	122.7 (6)
C9'—C10'—C14'	97.7 (11)	C40—C41—C42	117.4 (6)
C10'—O2'—H2'	109.5	C41—C42—H42A	109.5
C35—C14'—C10'	98.8 (11)	C41—C42—H42B	109.5
C35—C14'—H14C	112.0	H42A—C42—H42B	109.5
C10'—C14'—H14C	112.0	C41—C42—H42C	109.5
C35—C14'—H14D	112.0	H42A—C42—H42C	109.5
C10'—C14'—H14D	112.0	H42B—C42—H42C	109.5
